# BMP-2 releasing mineral-coated microparticle-integrated hydrogel system for enhanced bone regeneration

**DOI:** 10.3389/fbioe.2023.1217335

**Published:** 2023-08-10

**Authors:** Hongwei Xu, Huanhuan Luo, Jiayu Chen, Gang Chen, Xiaohua Yu, Zhaoming Ye

**Affiliations:** ^1^ Orthopaedic Oncology Services, Department of Orthopaedics, The Second Affiliated Hospital of Zhejiang University School of Medicine, Hangzhou, China; ^2^ Jiaxing Key Laboratory of Basic Research and Clinical Translation on Orthopedic Biomaterials, Department of Orthopaedics, The Second Affiliated Hospital of Jiaxing University, Jiaxing, China; ^3^ Orthopaedic Research Institute, Zhejiang University, Hangzhou, China; ^4^ Key Laboratory of Motor System Disease Research and Precision Therapy of Zhejiang Province, Hangzhou, China; ^5^ The Second Clinical Medical College of Zhejiang Chinese Medical University, Hangzhou, China

**Keywords:** large bone defects, hydrogel, mineral coating, bone morphogenetic protein-2, controllable release

## Abstract

**Introduction:** Large bone defects (LBD) caused by trauma, infection, and tumor resection remain a significant clinical challenge. Although therapeutic agents such as bone morphogenetic protein-2 (BMP-2), have shown substantial potency in various clinical scenarios, their uncontrollable release kinetics has raised considerable concern from the clinical viewpoint. Mineral-coated microparticle (MCM) has shown its excellent biologics loading and delivery potential due to its superior protein-binding capacity and controllable degradation behaviors; thus, it is conceivable that MCM can be combined with hydrogel systems to enable optimized BMP-2 delivery for LBD healing.

**Methods:** Herein, BMP-2 was immobilized on MCMs via electrostatic interaction between its side chains with the coating surface. Subsequently, MCM@BMP-2 is anchored into a hydrogel by the crosslinking of chitosan (CS) and polyethylene glycol (PEG).

**Results and Discussion:** This microparticle–hydrogel system exhibits good biocompatibility, excellent vascularization, and the sustained release of BMP-2 in the bone defect. Furthermore, it is observed that this microsphere–hydrogel system accelerates bone formation by promoting the expression of osteogenesis-related proteins such as RUNX2, osteopontin, and osteocalcin in bone marrow mesenchymal stem cells (BMSCs). Thus, this newly developed multifunctional microparticle–hydrogel system with vascularization, osteogenesis, and sustained release of growth factor demonstrates an effective therapeutic strategy toward LBD.

## Introduction

Large bone defect (LBD) is a complex disease, with the leading incidence among patients who have experienced trauma, infection, and tumor resection (Vidal et al., 2020). Typically, autologous bone transplantation or allogeneic bone transplantation is considered an effective approach to treat LBD (Liu et al., 2013; Tang et al., 2020). However, autologous bone transplantation suffers from lack of donors and the pain associated with secondary surgery (Azi et al., 2016), and allogeneic bone transplantation is prone to a range of problems for patients such as disease transmission and immune rejection (Hofmann et al., 1995). In recent years, growth factor therapeutics have attracted significant attention (Agarwal and García, 2015; Hu and Olsen, 2016). However, their uncontrollable release kinetics severely inhibits their development (Cui et al., 2020). Therefore, it is urgent to explore a facile scaffold strategy to establish potent therapeutics against LBD.

Tissue engineering is a promising way to deal with LBD (Turnbull et al., 2018; Abdollahiyan et al., 2020; Meng et al., 2022). Hydrogels have received extensive attention in bone repair because of good biocompatibility and retention (Agarwal and García, 2015; Yue et al., 2020; Yue et al., 2020). For instance, Wu et al. prepared hydrogel microspheres based on photo-cross-linked gelatin hydrogels, promoting regeneration of cancellous bone (Wu et al., 2020). Wang et al. employed chitosan hydrogel to deliver vascular endothelial growth factor and bone morphogenetic protein-2 (BMP-2) for the treatment of mandibular defects in rabbits, achieving sustainable release and bioactivity preservation for the growth factors (Wang et al., 2020). Therefore, hydrogels hold great promise as an excellent tissue engineering scaffold in the treatment of bone injuries.

However, hydrogels easily absorb a large amount of water in interstitial fluid, resulting in rapid release of protein, which in turn reduces the retention of protein at injury (Schloss et al., 2016; Wei et al., 2019; Bai et al., 2020; Zhang et al., 2023). Therefore, there is an urgent need to explore a complex drug delivery system to prolong the retention of proteins and promote their sustained release. Mineral-coated microparticle (MCM) is a great drug delivery system for growth factors (Yu et al., 2014; Yu et al., 2017; Bjelić and Finšgar, 2021), which is obtained by the surface modification of the microsphere, followed by immersion in modified simulated body fluid (mSBF) (Suárez-González et al., 2012). The coating of the MCM possesses excellent biocompatibility, biodegradability, and plenty of nanostructures formed by crystal growth. More importantly, since the coating contains a large amount of calcium and phosphate ions, it has a strong affinity for various biological macromolecules, which can be used as a multifunctional biomolecule delivery system for tissue repair (Lee et al., 2011; Baino and Yamaguchi, 2020). However, the MCM in bone defects is prone to be quickly metabolized with the circulation of tissue fluids. Thus, it will be of great importance to construct novel carriers such as microsphere–hydrogel complexes for the stable accumulation and effective release of growth factors in LBD.

**SCHEME 1:**
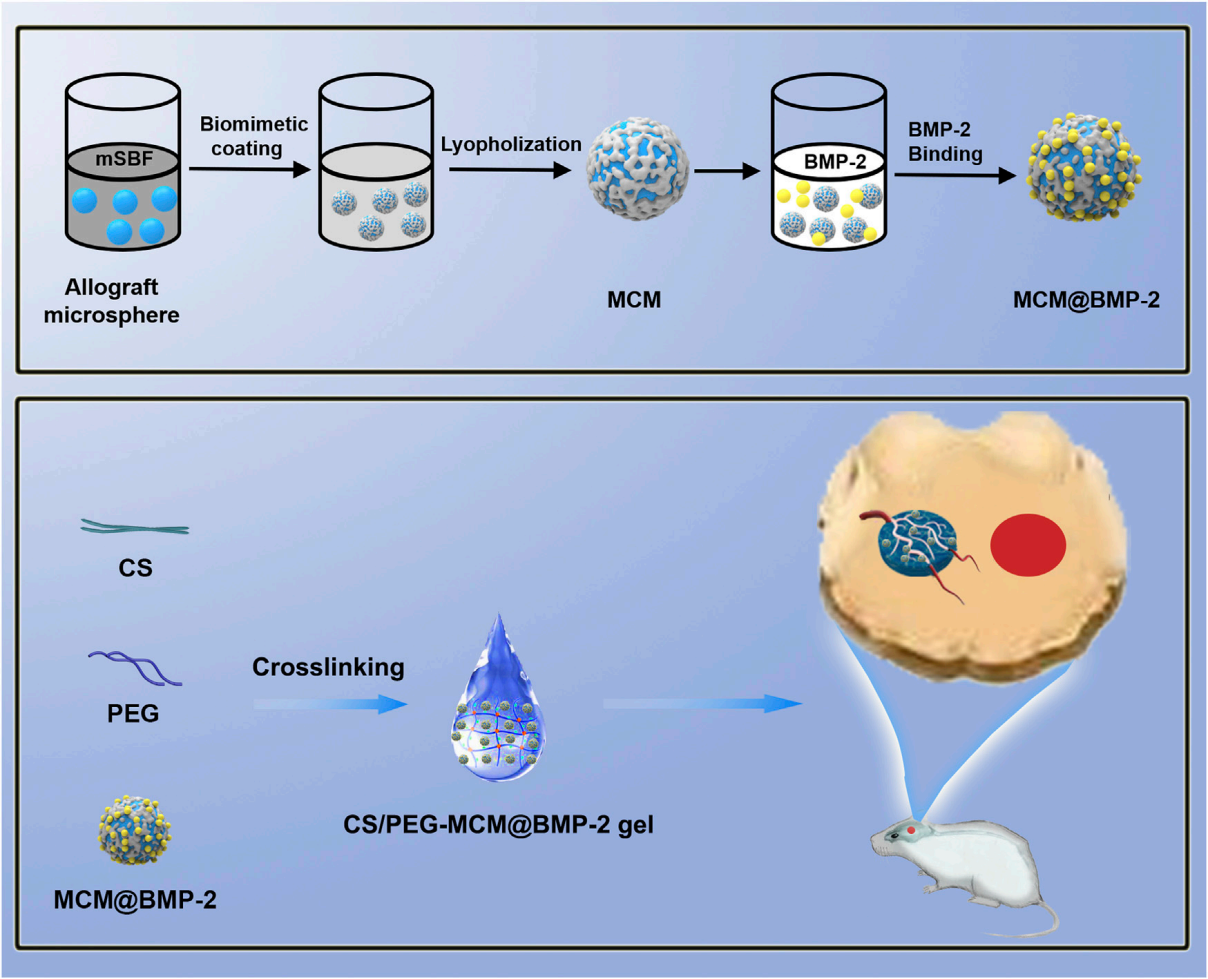
Schematic illustration of the composite hydrogel assembly and large bone defect repairing.

Herein, a composite hydrogel scaffold system of microsphere-hydrogel was successfully constructed by the electrostatic interaction between the side chains of BMP-2 with the coating surface of MCM, followed by the anchorage of MCM@BMP-2 in chitosan/polyethylene glycol (CS/PEG) hydrogel, achieving effective repair in LBD (Scheme 1). This composite hydrogel (CS/PEG-MCM@BMP-2 gel) provided an environment or space for new bone to grow, and the pore size of the hydrogel scaffold also provides an exchange space for the nutrients required for osteogenesis. Moreover, the MCM inside could promote sustained-release behavior of BMP-2 and produce a large number of calcium and phosphate ions to promote osteogenesis. Interestingly, this scaffold also promotes the tube-forming behavior of endothelial cells and repairs the formation of the vascular network. Thus, we expect that this innovative composite microsphere–hydrogel scaffold will have a significant application value in the treatment of LBD.

## Results and discussion

### Preparation and characterization

**FIGURE 1:**
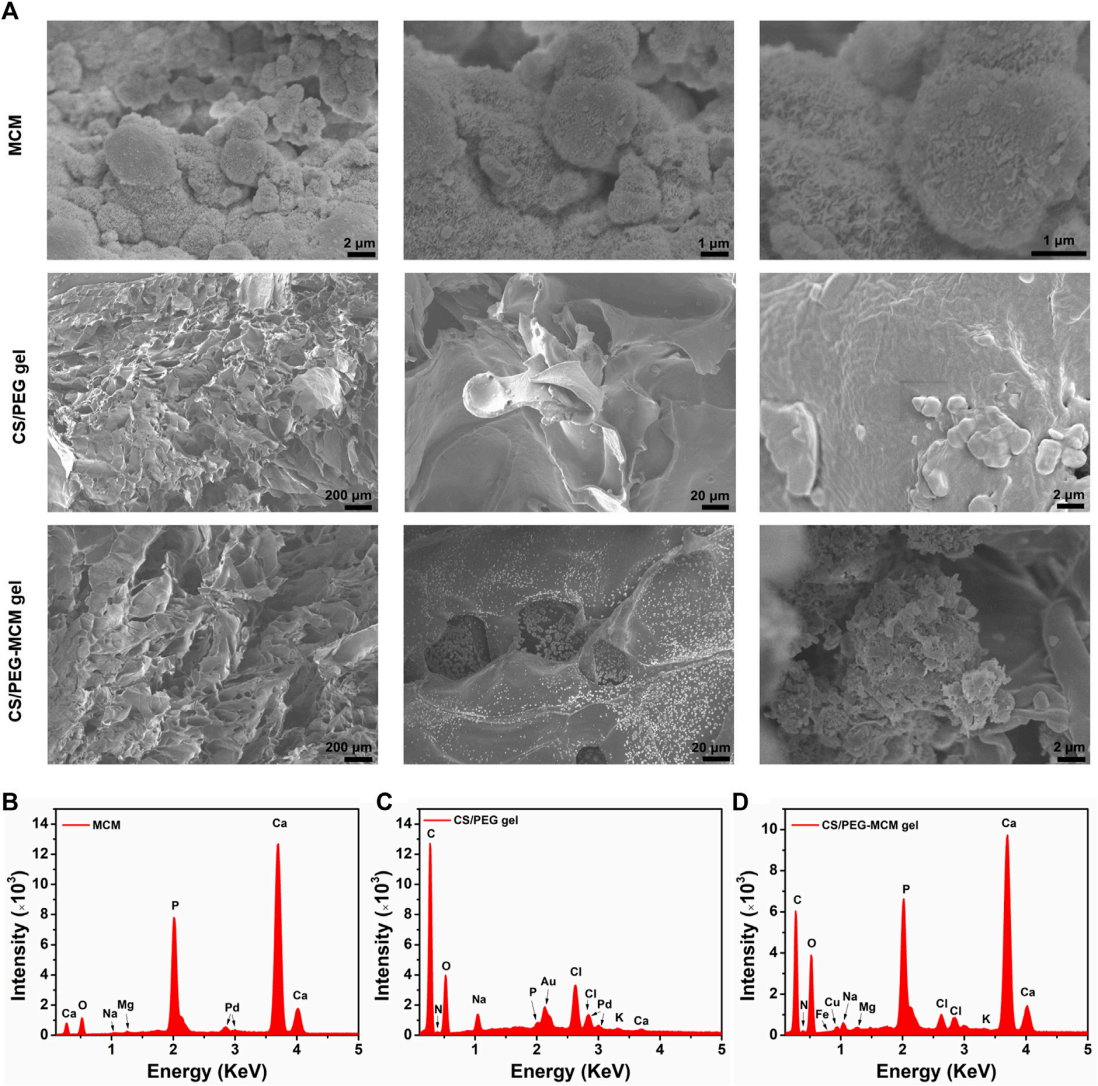
Morphology and element analysis. **(A)** SEM image of the MCM, CS/PEG gel, and CS/PEG-MCM gel at different scale bars. **(B–D)** EDS analysis of the MCM, CS/PEG gel, and CS/PEG-MCM gel.

Allograft microspheres that could induce autologous bone regeneration have been widely applied in clinical trials (Krauss, 1999). In this research, allograft microspheres were immersed in mSBF for surface mineralization to form nanostructured mineral coatings (Scheme 1). After mixing PEG with CS, the CS/PEG gel was formed by the cross-linking effect. Compared with the CS/PEG gel, CS/PEG-MCM had a rougher surface due to the presence of MCM. As shown in Figure 1A, MCMs were uniformly covered by a layer of coating with a typical plate-like structure after immersion in mSBF, and the surface of the MCMs was determined as having significant amounts of calcium and phosphorus by energy-dispersive spectroscopy (EDS) (Figure 1B), which was helpful for the loading of protein and promoting osteogenesis. CS has been widely used in biomedical fields because of its good biodegradability and excellent cell affinity. After mixing the CS solution with the PEG solution, the hydrogel (CS/PEG gel) was formed by the cross-linking effect. A uniform network structure was observed using a scanning electron microscope (SEM). Then, MCMs were dispersed with the PEG solution, followed by cross-linking with CS for the generation of the CS/PEG-MCM gel. As shown in Figure 1A, there is a big difference between the CS/PEG gel and the CS/PEG-MCM gel. The surface of the CS/PEG gel was relatively smooth, while that of the latter was covered with many microspheres. Furthermore, the results of EDS further demonstrated that MCM was successfully embedded in the hydrogel because the intensity of calcium and phosphorus in the CS/PEG gel (Figure 1C) was much lower than that in the CS/PEG-MCM gel (Figure 1D).

**FIGURE 2:**
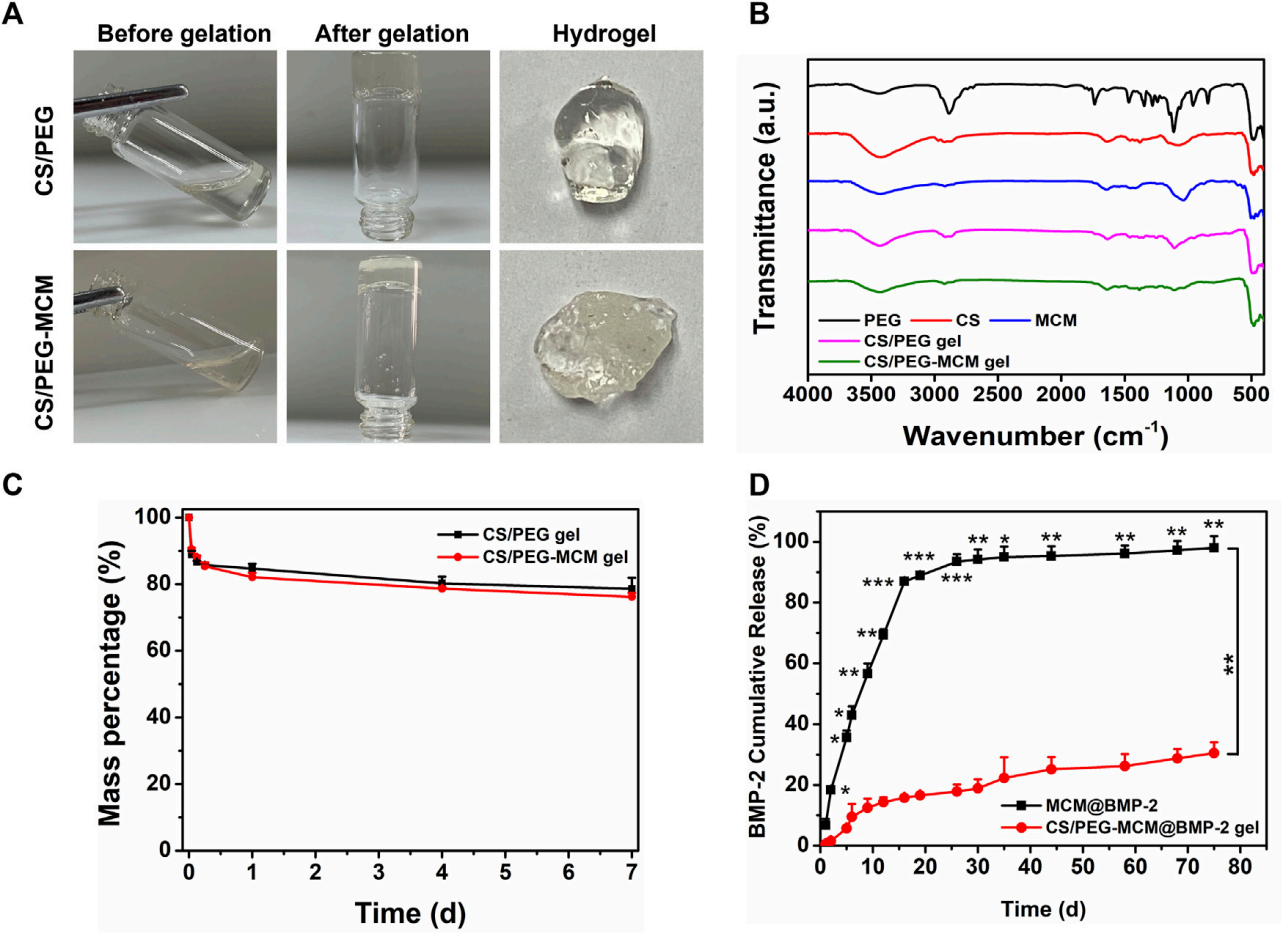
Preparation and characterization. **(A)** Preparation of the CS/PEG gel and CS/PEG-MCM gel. **(B)** FTIR spectrum of various samples. **(C)** Degradation of the CS/PEG gel and CS/PEG-MCM gel in PBS. **(D)** Release behavior of BMP-2 from the MCM@BMP-2 and CS/PEG-MCM@BMP-2 gel in PBS (a two-way ANOVA was used to analyze the difference between the MCM@BMP-2 and CS/PEG-MCM@BMP-2 gel, and multiple comparison (Student’s t-test) tests were performed between groups at each time point individually).

Then, in order to further demonstrate the successful preparation of the CS/PEG gel and the loading of MCM in the hydrogel, the gelation process of the hydrogel was observed (Figure 2A). Before gelation, both the mixtures of CS/PEG and CS/PEG-MCM had excellent flowability. After approximately 4 min of the reaction, the solution transformed into solid hydrogel, which did not flow as the bottle was turned upside down. In addition, the surface of the CS/PEG-MCM gel was much rougher than that of the CS/PEG gel due to the presence of MCM. Next, we further examined the Fourier transform infrared (FT-IR) spectrum of the various samples. The absorption peak of CS at 2,970 cm^−1^ had disappeared in the CS/PEG gel, indicating the chemical reaction between CS and PEG, which may be the mechanism of the cross-linking of the hydrogel. Compared with the CS/PEG gel, the absorption peak of the CS/PEG-MCM gel around 1,110 cm^−1^ had significantly decreased, indicating that MCM was successfully introduced to the hydrogel (Figure 2B).

### Degradation and release behavior

Subsequently, to investigate the degradation of the hydrogel, the CS/PEG gel and CS/PEG-MCM gel were soaked in PBS for different time periods. As shown in Figure 2C, both degraded quickly within 6 h, and then the degradation tended to be slow. They had only lost about 20% after 1 week, which indicates their superb retention ability in defects. Then, in order to determine the sustained-release ability of the composite hydrogel, the release behavior of BMP-2 in MCM@BMP-2 and CS/PEG-MCM@BMP-2 was observed. As shown in Figure 2D, the cumulative release of BMP-2 from the MCM@BMP-2 gel was 86.9% ± 0.7% on day 16 and continued increasing to 98.0% ± 3.7% on day 75. As for the CS/PEG-MCM@BMP-2 gel, the cumulative release of BMP-2 was only 30.5% ± 3.6% on day 75. Thus, it is obvious that the sustained-release effect of the composite hydrogel was much better than that of MCM, which exhibited a promising strategy in tissue engineering.

### Biocompatibility *in vitro* and tube formation abilities

**FIGURE 3:**
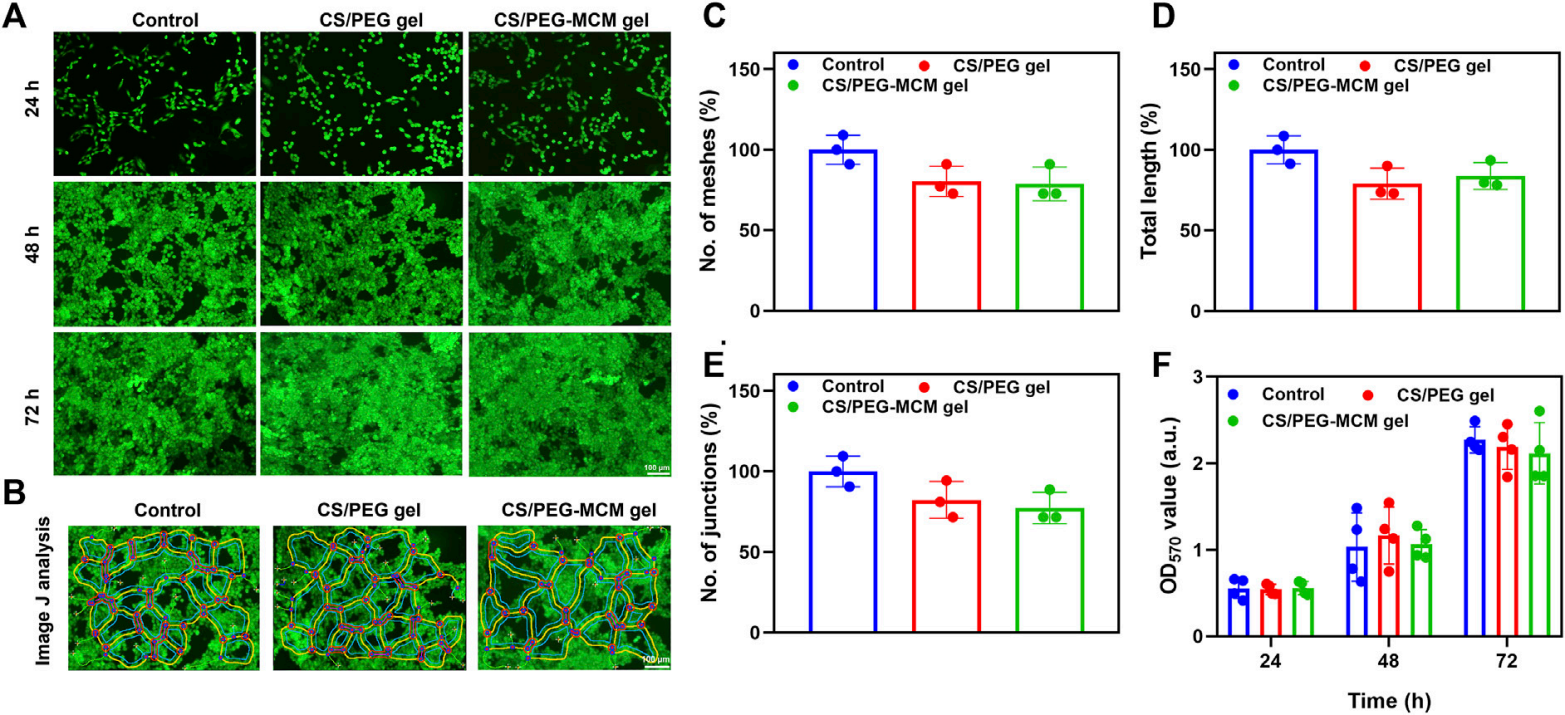
Biocompatibility analysis. **(A)** Live/dead staining of control, CS/PEG gel, and CS/PEG-MCM gel after 24, 48, and 72 h of incubation with HUVECs. **(B)** ImageJ analysis of the tube-forming behavior of HUVECs incubated with control, the leaching solution of the CS/PEG gel, and the CS/PEG-MCM gel. **(C)** ImageJ analysis of meshes. **(D)** ImageJ analysis of total length. **(E)** ImageJ analysis of junctions. **(F)** Cell viability of the HUVECs treated with control, the leaching solution of the CS/PEG gel, and the CS/PEG-MCM gel. (The control group means the cells treated with DMEM containing 10% fetal bovine serum and 1% penicillin–streptomycin. A one-way ANOVA with Tukey’s multiple comparisons test was performed between groups, and they showed no significant difference.).

Live/dead staining (Sang et al., 2022) was used to determine the biocompatibility of the hydrogels. Calcein-AM can pass through the cell membrane and produce calcein that can emit green fluorescence under the action of intracellular enzymes, while PI as a nuclear dye cannot pass through the living cell membrane—it can only pass through the cell membrane of dead cells to the nucleus, react with DNA, and emit red fluorescence (Luo et al., 2021a; Luo et al., 2021b). In order to measure the biocompatibility of the hydrogel scaffolds, HUVECs were treated with the supernatant from the hydrogels for 24, 48, and 72 h. As shown in Figure 3A, all groups displayed a good morphology at 24 h, while they still maintained a favorable proliferative ability at 48 and 72 h. In addition, there was no significant difference among the three groups, and they all exhibited excellent proliferative activity and showed green fluorescence, which indicates the good biocompatibility of the hydrogel. Interestingly, HUVECs treated with these hydrogel extracts seemed to undergo tube formation after 48 h of incubation. Then, we employed ImageJ to analyze the pictures (Figure 3B). To our surprise, all of them possessed the ability to promote tube formation, and there was no significant difference in meshes, total length, and junctions (Figures 3C–E). Then, we further performed an MTT assay in HUVECs to quantitatively detect the cell proliferation ability. In addition, the optical density (OD) values were also similar, and the statistics had no difference. In addition, the OD values were increased over time, which further demonstrated the superior biocompatibility of the hydrogels. Thus, both results showed good compatibility of the hydrogels.

### *In Vitro* osteogenic differentiation

**FIGURE 4:**
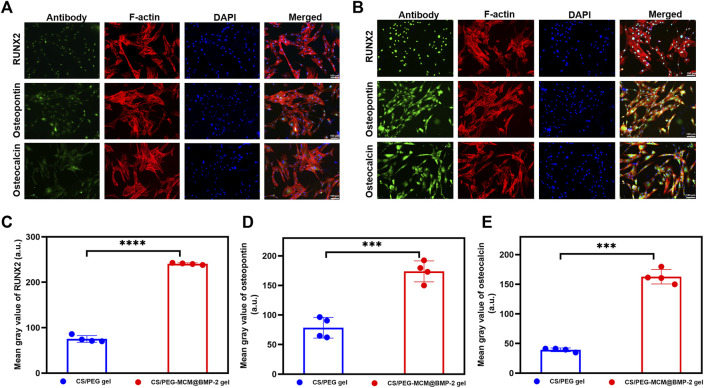
Immunofluorescence analysis *in vitro*. **(A)** Fluorescent images of RUNX2, osteopontin, and osteocalcin proteins in BMSCs treated with the leaching solution of the CS/PEG gel. **(B)** Fluorescent images of RUNX2, osteopontin, and osteocalcin proteins in BMSCs treated with the leaching solution of the CS/PEG-MCM@BMP-2 gel. **(C)** ImageJ analysis of the expression of RUNX2. **(D)** ImageJ analysis of the expression of osteopontin. **(E)** ImageJ analysis of the expression of osteocalcin. (Student’s t-test was performed between groups.).

In order to verify the osteogenic differentiation ability of the hydrogel, we performed an immunofluorescence experiment to study osteogenic-related protein expression in BMSCs. RUNX2 is a specific transcription factor for osteoblasts and plays an important role in bone formation and reconstruction. It determines the differentiation of pluripotent stem cells to osteoblasts and promotes chondrocyte maturation and cartilage vascularization (Komori, 2019). In addition, osteopontin is an important bone matrix protein, which is closely related to bone formation and development (Si et al., 2020). As for osteocalcin (Komori, 2020), it is mainly synthesized by osteoblasts and dentin cells, which play an important role in regulating bone calcium metabolism. Therefore, these osteogenic proteins play an important role in osteogenic differentiation. In order to have a more accurate image, F-actin (red) was used to observe the cytoskeleton of the cells, and DAPI (blue) was used as a dye to observe the nucleus. As shown in Figures 4A, B, the cells treated with both the CS/PEG gel and CS/PEG-MCM@BMP-2 gel had a good morphology, which further demonstrated the good biocompatibility of the hydrogels. However, the intensities of the green fluorescence of RUNX2, osteopontin, and osteocalcin in the CS/PEG-MCM@BMP-2 gel were all stronger than that in the CS/PEG gel. In addition, according to the results of ImageJ analysis, the expressions of RUNX2 (Figure 4C), osteopontin (Figure 4D), and osteocalcin (Figure 4E) in the cells treated with CS/PEG-MCM@BMP-2 gel were all higher than that in the CS/PEG gel, which indicated that this composite hydrogel greatly supported osteogenic differentiation.

### Bone defect regeneration *in vivo*

**FIGURE 5:**
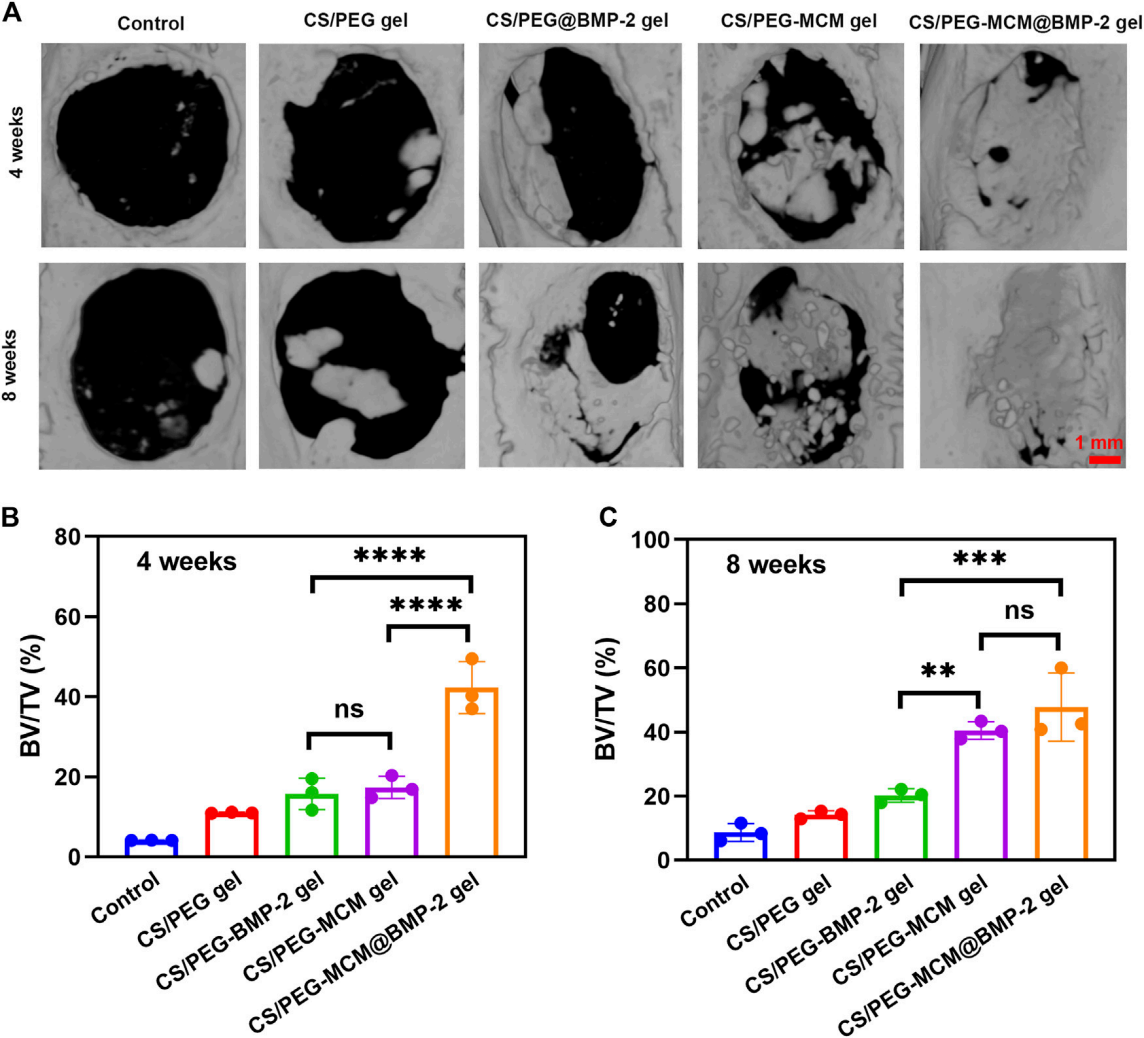
3D reconstruction. **(A)** Micro-CT image of the cranium harvested from SD rats after treatment with various hydrogels at weeks 4 and 8. **(B)** Bone volume/tissue volume (BV/TV) analysis at 4 weeks. **(C)** Bone volume/tissue volume (BV/TV) analysis at 8 weeks. (A one-way ANOVA with Tukey’s multiple comparisons test was performed between groups.).

To investigate the ability of the composite hydrogel to reconstruct bone defects *in vivo*, a rat cranium defect model was constructed by electric drill after anesthesia, and then the various hydrogels were implanted into the defects, followed by the suture. After rearing for 4 and 8 weeks, the skulls were extracted from the rats and immersed in paraformaldehyde solution for further determination. In order to intuitively observe the regeneration of the skull in rats, micro-computed tomography (micro-CT) was performed. From the 3D reconstruction of the cranium defect by micro-CT (Figure 5A), the new bone coverage areas of the same samples at 8 weeks were all higher than those at 4 weeks, which indicated that these hydrogels all had some bone regeneration ability, but there was a difference in efficacy, which indicated the sustained release of the hydrogel and a little self-healing ability. The CS/PEG gel had the weakest efficacy, whether at week 4 or 8, the new bone coverage area was the smallest. The CS/PEG@BMP-2 gel and CS/PEG-MCM gel had a better ability in bone regeneration, which may be contributed to the presence of BMP-2 or MCM in the hydrogel, respectively. Most importantly, the CS/PEG-MCM@BMP-2 gel was clearly higher than other hydrogels at 4 weeks (Figure 5B). However, at week 8, the fracture of new bone in defects of CS/PEG-MCM@BMP-2 had no significant difference from that of CS/PEG-MCM, which may be due to the presence of MCM or the sustained release of BMP-2 (Figure 5C), which requires further exploration.

### Histological analysis

**FIGURE 6:**
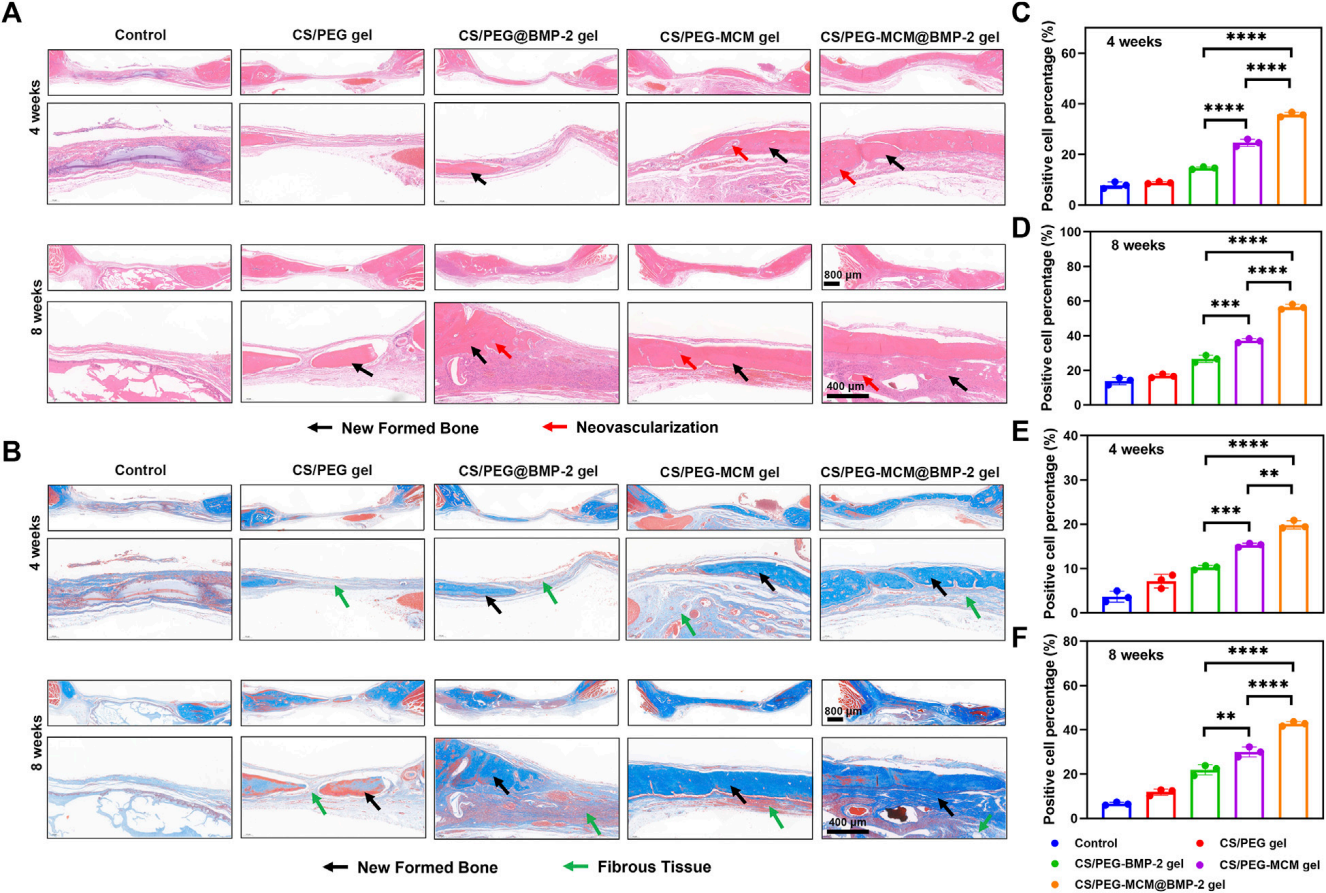
Immumohistochemical staining. **(A)** HE staining of tissue sections of the cranium harvested from SD rats after treated with the hydrogel at weeks 4 and 8. **(B)** Masson staining tissue sections of the cranium harvested from SD rats after treated with the hydrogel at weeks 4 and 8. **(C)** ImageJ analysis of HE staining at week 4. **(D)** ImageJ analysis of HE staining at week 8. **(E)** ImageJ analysis of masson staining at week 4. **(F)** ImageJ analysis of Masson staining at week 8. (A one-way ANOVA with Tukey’s multiple comparisons test was performed between groups.)

In order to further explore the osteogenic effect of the hydrogels, the cranium tissues of various samples at 4 and 8 weeks were harvested from the rats for hematoxylin and eosin (HE) staining to evaluate the formation of new bone (Miao et al., 2019). The pink part in the middle of HE staining image indicated the formation of the new bone. As illustrated in Figure 6A, the new bone coverage area of the CS/PEG-MCM@BMP-2 gel was 35.7% ± 0.9% (Figure 6C) at 4 weeks and continued increasing to 56.5% ± 1.5% (Figure 6D) at 8 weeks, both of which had a significant difference from other groups. Then, in order to further determine the ability of bone regeneration, Masson staining was also employed (Cheng W. et al., 2020). As reported earlier, the newborn collagen fibers can react with aniline blue to appear blue, and tendon fibers can react with acid fuchsin to appear red. In addition, the new bone is rich in collagen, which indicates that the larger the blue area, the more bone is produced. As shown in Figure 6B, the CS/PEG-MCM@BMP-2 gel possessed the most area of new bone. After quantitative analysis by ImageJ, the percentage of the new bone in CS/PEG-MCM@BMP-2 both at 4 weeks (Figure 6E) and 8 weeks (Figure 6F) had a statistical difference with that of other hydrogels, which further demonstrated the better osteogenic ability of the CS/PEG-MCM@BMP-2 gel.

**FIGURE 7:**
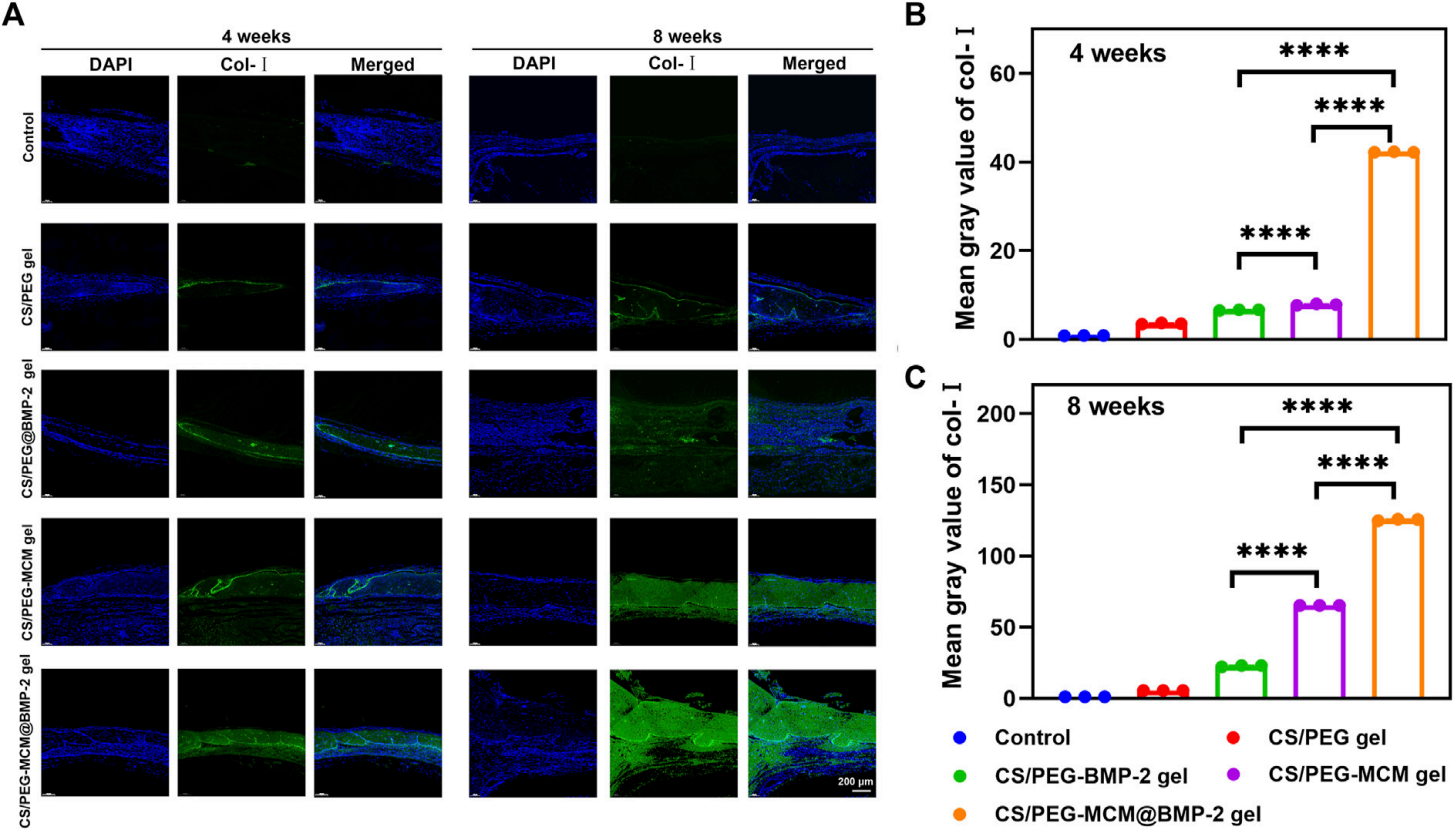
**(A)** Image of immunofluorescence staining of Col-Ⅰ. Col-Ⅰ in the cells was stained in green, and the nuclei were stained in blue. **(B)** ImageJ analysis of fluorescence intensity of Col-Ⅰ at week 4. **(C)** ImageJ analysis of fluorescence intensity of Col-Ⅰ at week 8. (A one-way ANOVA with Tukey’s multiple comparisons test was performed between groups.)

**FIGURE 8:**
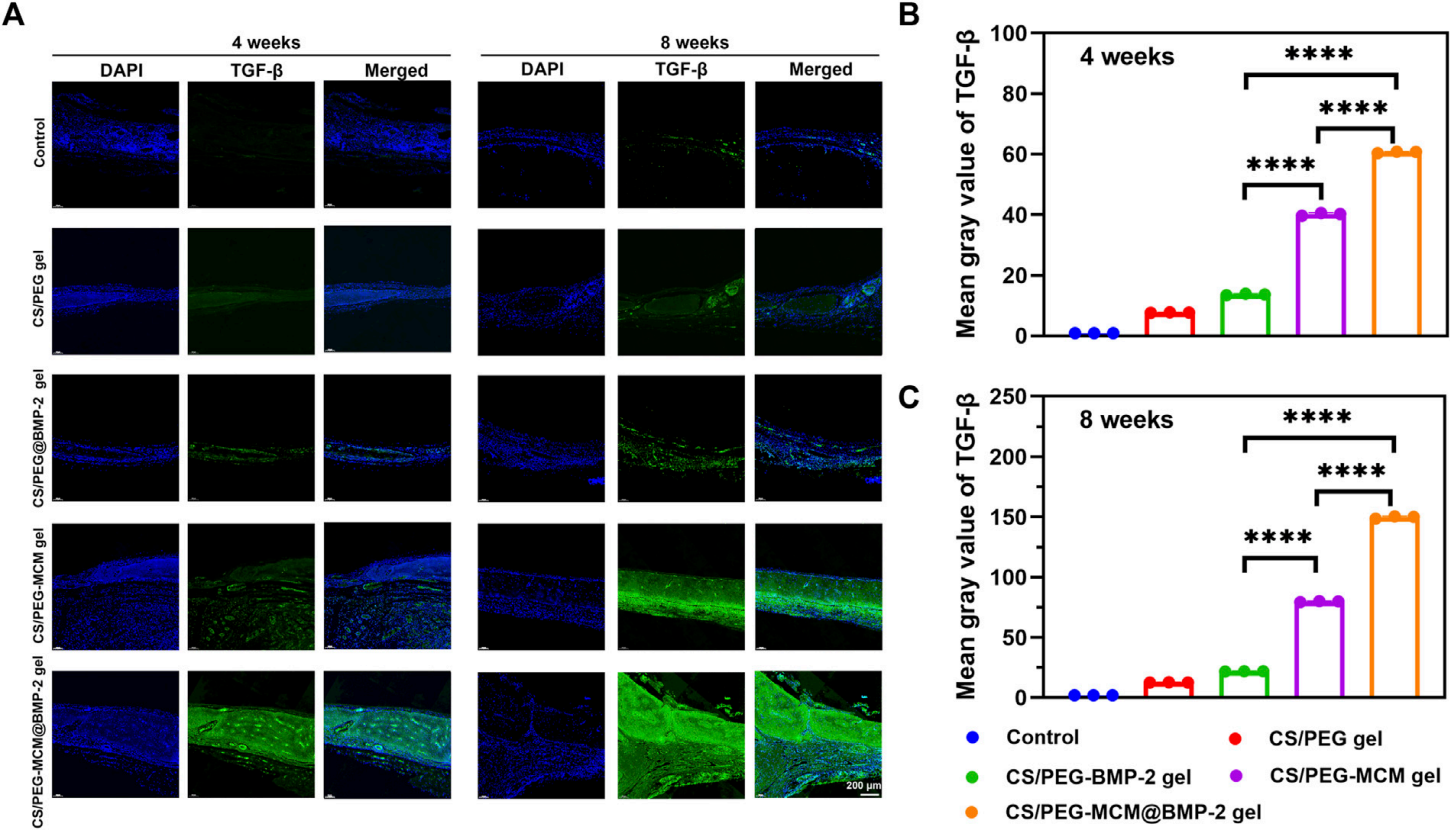
**(A)** Image of immunofluorescence staining of TGF-β. TGF-β in the cells was stained in green, and the nuclei were stained in blue. **(B)** ImageJ analysis of fluorescence intensity of TGF-β at week 4. **(C)** ImageJ analysis of fluorescence intensity of TGF-β at week 8. (A one-way ANOVA with Tukey’s multiple comparisons test was performed between groups.)

Subsequently, we further performed immunofluorescent staining to explore the expression of collage to clarify the osteogenic ability of the hydrogels. Col-Ⅰ is a significant marker of the formation of bone mechanical strength. With time, the expression of Col-Ⅰ was higher at 8 weeks than at 4 weeks (Figure 7A). Moreover, the tissues treated with the CS/PEG-MCM@BMP-2 gel showed the highest intensity of Col-Ⅰ at 8 weeks (Figures 7B, C). Furthermore, we also explored the expression of TGF-β by immunofluorescent staining (Figure 8A). TGF-β is a protein that plays an important part in bone formation. As shown in Figures 8B, C, the expression of TGF-β was also higher in the group of the CS/PEG-MCM@BMP-2 gel, which showed the same results as the expression of Col-Ⅰ. Thus, this composite hydrogel showed a good effect on bone defect repairing *in vivo*.

## Conclusion

In conclusion, we have successfully constructed a composite microsphere–hydrogel protein delivery system by the electrostatic interaction between BMP-2 and MCMs, followed by the anchorage of MCM@BMP-2 with the CS/PEG hydrogel for LBD therapy. The composite hydrogel CS/PEG-MCM@BMP-2 gel with osteogenesis and vascularization capacity exhibited unique degradation and excellent biocompatibility. In addition, the animal experiments further demonstrated the ability of the CS/PEG-MCM@BMP-2 gel to increase the formation of new bone *in vivo*. Thus, the versatile design of this composite microsphere–hydrogel scaffold possesses great potential in bone defect repair.

## Materials and methods

### The preparation of MCM

Allograft bone microsphere was purchased from Yapeng Biological. Briefly, 1 mg of allograft bone microspheres was immersed in 0.5 mL of SBF for a week for mineralization, and then the fresh SBF was added to replace the original solution every day. Finally, the MCM was collected after lyophilization.

### The preparation of the hydrogel

For the preparation of the CS/PEG gel, we first configured the solution of the pre-hydrogel. A total of 0.15 g of chitosan (CS) and 0.35 g of polyethylene glycol (PEG) were dissolved in 2 mL of PBS under ultrasonication. Then, the CS/PEG gel was prepared after 4 min of mixing the CS solution with the aforementioned PEG solution.

For the preparation of the CS/PEG-MCM gel, microspheres (allograft, Yapeng Biological) were immersed in mSBF for the generation of MCM. Then, 10 mg of MCM was dispersed in 50 μL PEG solution, followed by the mixture with 50 μL CS.

As for the CS/PEG-MCM@BMP-2 gel, 10 μg BMP-2 and 10 mg MCM were added together to 1 mL PBS at 37°C for 4 h incubation to allow the binding of BMP-2. Then, the aforementioned MCM@BMP-2 was dispersed in the PEG solution, followed by cross-linking with the CS solution.

### Characterization

The morphology and energy-dispersive X-ray spectroscopy (EDS) of the freeze-dried hydrogel and MCM were observed using a scanning electron microscope (SEM, HITACHI S-4800). The infrared spectrogram of the samples was determined by Fourier transform infrared spectroscopy.

### Degradation test

The degradation behavior of the hydrogel was determined in the PBS solution (pH 7.4) (Cheng et al., 2020a). First, the primary mass of the freeze-dried hydrogel was recorded. Then, the freeze-dried hydrogel was immersed in 2 mL PBS in an oscillator shaker with constant temperature (37°C). At each time point, the hydrogel was taken from the tube, followed by the recording the mass after being freeze-dried.

### Drug release

The drug release behavior of BMP-2 from MCM@BMP-2 or CS/PEG-MCM@BMP-2 was evaluated in the PBS solution (pH 7.4). The supernatants were taken from the solutions in an oscillator shaker with constant temperature (37°C) at various time points by centrifugation, and fresh PBS was added to the tubes for further immersion. Finally, the concentration of BMP-2 was determined using an ELISA kit.

### Biocompatibility test and tube-forming analysis

Human umbilical vein endothelial cells (HUVECs) were cultured in DMEM containing 10% fetal bovine serum (FBS) and 1% penicillin–streptomycin (PS) at 37°C under 5% CO_2_. For the biocompatibility test, live–dead staining was performed using a living/dead cell double staining kit (Luo et al., 2021b). Calcein-AM is able to easily penetrate into living cell membranes, followed by green fluorescence, which is only suitable for living cells. In addition, pyridine iodide (PI) is only used for dead cells for red fluorescence. HUVECs were seeded on a 24-well plate overnight, and the leaching solutions of the CS/PEG gel and CS/PEG-MCM gel were added to the plate. After 24-, 48-, and 72-h incubation, the mixture of 2 μM calcein-AM and 8 μM PI was added to the plate for 30 min of incubation. Then, the cells were washed and observed using a fluorescent microscope. In addition, the tube-forming behavior was analyzed by ImageJ software.

We further performed an MTT assay to determine the cell viability of the hydrogel. HUVECs were seeded on a 96-well plate overnight, and the leaching solutions of the CS/PEG gel and CS/PEG-MCM gel were added to the plate for further incubation. After 24-, 48-, and 72-h incubation, the cells were washed, and the MTT solution was added for 4 h of incubation. Finally, the cell viability was measured by the addition of DMSO, followed by the measurement using a microplate reader.

### Immunofluorescence analysis

Bone marrow mesenchymal stem cells (BMSCs) wer cultured in α-MEM containing 10% FBS and 1% PS at 37°C under 5% CO_2_. First, BMSCs were seeded on a glass in a 6-well plate in a cell incubator overnight, and the leaching solutions of CS/PEG gel and CS/PEG-MCM@BMP-2 gel were added into the plate for 24 h of incubation. In addition, the cells were washed and incubated with 4% paraformaldehyde for 20 min at 37°C. Next, the cells were washed with PBS three times, and 0.5% Triton X-100 was added to the plate for 20 min of incubation at room temperature. After washing with PBS three times again, the cells were incubated with 5% BSA for 30 min for closure. Then, osteogenic antibodies including RUNX-2, osteopontin, and osteocalcin were added to the plates, followed by further incubation at 4°C overnight. The next day, the glasses were washed with PBST three times, followed by 1 h of incubation with goat anti-rabbit IgG-H&L at room temperature. Then, DAPI was added to the plate for 5 min of incubation. Finally, the glasses were washed with PBST and observed using a fluorescent microscope (Miao et al., 2019). The results were analyzed by ImageJ.

### LBD repair

A rat skull defect model was constructed for the research model (Cheng et al., 2020b). The CS/PEG gel and CS/PEG-MCM@BMP-2 gel were implanted into the skull defect for a certain period of time. Then, the area of the skull was detected using an X-ray image analysis system and extracted from the rat for further immunohistochemical analysis.

### Immunohistochemical analysis

The skull extracted from the rat was fixed in 4% polyformaldehyde for 24 h, followed by decalcification with 10% EDTA for 1 week at 37°C. Then, the samples were embedded in paraffin and sliced into 5-μm-thick sections. Finally, the slides were reacted with hematoxylin and eosin, Masson, Col-Ⅰ, and TGF-β (Qiao et al., 2020).

### Statistical analysis

All data were reported as mean ± standard deviation (SD), and the data were analyzed by the GraphPad software (ns, non-significance, *p < 0.05, **p < 0.01, ***p < 0.001, and ****p < 0.0001).

## Data Availability

The original contributions presented in the study are included in the article/Supplementary Material; further inquiries can be directed to the corresponding authors.

## Abbreviations

LBD, large bone defects; BMP-2, bone morphogenetic protein-2; MCM, mineral-coated microparticle; CS, chitosan; PEG, polyethylene glycol; BMSC, bone marrow mesenchymal stem cell; mSBF, modified simulated body fluid; SEM, scanning electron microscope; FTIR, Fourier transform infrared; micro-CT, micro-computed tomography; HE, hematoxylin and eosin.
